# Shocked into Silence

**Published:** 1892-10

**Authors:** 


### SHOCKED INTO SILENCE.

We clip the following pathetic account from a recent issue of that stanch humanitarian sheet, "The Banner of Light," which is always to be found on the side of the oppressed and suffering of whatever race or country.

It is shameful enough that such fiendish brutality and tenderness are ever suffered to be brought into contact, even in our prisons, and something is decidedly wrong in the government of such as permit it.

How often it is that a kind and loving soul, in a moment of thoughtless desperation, is unhappily brought within the corrective system of our criminal code, and shame-faced and repentant, shrinks from the sting of an awakened conscience as from a viper, till overcome by the sweet memory of brighter days, finds vent in song, that in itself is a redeeming grace, only to be interrupted by the blasphemous oath of some brute, lost to himself and all mankind, whose place should be beyond the possibility of obtrusion.

But to the sketch in question :

On a certain superlatively beautiful morning recently, the fragrance of the new-mown hay in a certain jail yard seemed to inspire all the prisoners with one common thought, one universal yearning for the free sun and air of heaven. Along all the corridors, from out the close cells, as if from the animation of a common impulse, rose the low, half-suppressed hum of voices in song. Every prisoner seemed to feel the benign influence of God's beauteous handiwork about him in sky and air, field and tree. Every heart seemed attuned to song, and all the songs were plaintively suggestive of some unsatisfied longing.

One voice, a girl's, tearful in its smothered sadness, seemed to echo the soft refrain of the dove, as the notes of "My Old Kentucky Home" floated forth from her lips. Another voice poured out in touching tenderness the air of "Down on the Farm." Another prisoner sang with a pathos the listener confessed he had never known before, "Oh, for a Closer Walk with God," as if there was a soulful longing for a closer communion with the purer spirits, the guardian angels of the world to come. With similar feelings, another was humming "Nearer, my God, to Thee." Still another plaintively sang "I would not live alway." Altogether it was a scene and sound never to be forgotten. One moment thus spent does more to purify the heart, reform the evil nature, and sow the seeds of good resolves, than weary months of punishment in the loneliness of a cell.

But even this poor solace of humming an air that wafted from the vanished years of childhood one fond memory of a better life, was denied the songful prisoners. The gruff, harsh voice of a profane "trusty" was soon heard, commanding the prisoners to "knock off that noise." A dead silence at once fell on all. But it seemed to be not the silence of a willing acquiescence, but of despair. As if it were too great a lightening of punishment to let the prisoners have a morning and an evening hour to sing or hum the songs that reach the heart and restore for the time the severed communion with all that is brighest and best, purest and noblest in their lives—lives too often darkened by sin and want, suffering and sorrow.

It is enough for a tender heart and loving nature to be shut out from the free sun and air, to see no more the tender light in the tear-dimmed eyes of loved ones, to hear no more the soft words that thrill the soul, to feel no more the magnetic touch of hands outstretched in sympathy, to feel athwart the heart the fell shadow of humiliation and disgrace, often unmerited, and to see, day by day, the sands of a brief existence swiftly pouring from the upper to the lower bulb of the hour glass.

Then let us not be to severe on the unfortunate prisoner. If all other links that bind us to the sweet past must be broken, and the fading of life's dreams must finally come to his eyes, let us at least span the thickening twilight for him with a bridge, not of sighs merely, but of that unbidden song that tells the sympathetic ear the tension of the heart-strings, as the notes of the harp show the strain upon its chords. It will do harm to no one, and who knows the good it may work, in the heart now and in the life hereafter.

What specially impresses the foregoing sketch, but briefly outlined, upon the sympathetic reader's heart, is the fact that it was written by a prisoner.

